# Fascia Lata Biological Plug: A Novel Technique for Treating Anal Fistulae

**DOI:** 10.7759/cureus.75437

**Published:** 2024-12-10

**Authors:** Ahmed Moustafa, Amr K Ebrahim, Ramy Saad, Osama R Mohamed, Mohamed Elbarmelgi, Hany A Balamoun, Ismail A Shafik

**Affiliations:** 1 General Surgery, Cairo University, Cairo, EGY; 2 Surgery, Maidstone and Tunbridge Wells NHS Trust, Maidstone, GBR

**Keywords:** biological plug, fascia lata, high anal fistula, incontinence, recurrence

## Abstract

Background: An anal fistula is a prevalent condition characterised by an abnormal connection between the epithelialised surface of the anal canal and the skin. Surgeons are continually developing new techniques to effectively treat anal fistulae while preserving the patient's continence. This study aims to evaluate the outcomes and complications associated with the management of high perianal fistulae using the fascia lata biological plug (FBP) technique.

Methods: This prospective cohort study included all adult patients who presented to the Kasr Al-Ainy Outpatient Surgery Clinic, Cairo, Egypt, between March 2020 and December 2021, with a single-tract high perianal fistula.

Results: A total of 46 eligible patients were included in the study. The insertion of the FBP was associated with complete healing without recurrence in 37 patients (80.4%) at six months post-surgery. Among the 46 patients, only nine (19.6%) experienced fistula recurrence. The recurrence rate increased to 30.4% at 18 months post-surgery, resulting in an overall success rate of 69.6%. Complete continence was maintained in all patients. At 18 months, extra-sphincteric (14.3% vs 0.0%) and supra-sphincteric (21.4% vs 0.0%) types exhibited significantly higher recurrence rates (p=0.006). Anterior fistulae also demonstrated a significantly higher recurrence rate compared to posterior fistulae (64.3% vs 25.0%, p=0.011).

Conclusions: The use of a FBP for the treatment of single-tract high perianal fistulae yields promising results without compromising patients' continence. It is essential to consider the type and nature of the anal fistula when selecting the most appropriate procedure for effective treatment.

## Introduction

An anal fistula is an abnormal connection between the epithelialised surface of the anal canal and the skin, representing one of the most common conditions affecting the anus. The primary cause of an anal fistula is infection and abscess formation. Inadequate drainage or spontaneous rupture of the abscess results in fistula formation. Less frequently, fistula formation can occur secondary to inflammatory bowel diseases such as Crohn's disease, haemorrhoidal surgery, chronic anal fissures, malignancy, or radiation therapy [1].

According to Park's classification, anal fistulae are categorised into inter-sphincteric, trans-sphincteric, supra-sphincteric, and extra-sphincteric types. The choice of treatment depends on the location of the fistula tract and the position of the internal opening within the anal canal [2].

Treatment for perianal fistulae is predominantly surgical, aiming to eradicate the infection and cure the fistula while preserving the anal sphincter. The dual objectives of resolving the fistula and maintaining continence present a significant challenge for surgeons, as fistulous tracts can encircle varying amounts of the sphincter complex [3]. Different surgical techniques have been developed, including fistulotomy, fistulectomy, advancement of mucosal flaps, and cutting setons. Although these conventional approaches have achieved success rates of up to 90%, they may lead to post-operative anal dysfunction due to potential damage to the anal sphincter [4]. To improve functional outcomes, several sphincter-sparing techniques have been introduced, including the use of platelet-rich plasma, fibrin adhesive glue, and anal fistula plug (AFP) insertion [5].

The AFP has emerged as a promising alternative to open surgery for treating anal fistulae. First described in 2006, it has demonstrated a significantly higher healing rate compared to fibrin glue [6]. Several studies have shown that the AFP is characterised by its simplicity of operation, minimal pain, preservation of the anal sphincter, and repeatable application [7-9]. However, variability in fistula anatomy, differences in inclusion criteria, and varying endpoints have complicated the assessment of the efficacy of these plugs [10].

The AFP is composed of biological materials, including acellular dermal matrix and submucosa derived from the porcine small intestine (Surgisis fistula plug (Biodesign, Cook Medical, Bloomington, IN), as well as new bioabsorbable synthetic materials (BIO-A fistula plug, Newark, DE) [7,8,11]. The fascia lata, a fascial layer surrounding the deep tissues of the thigh, has been utilised for repairing urethral fistulae [12] and in robotic reconstructive surgery of the genitourinary tract [13]. We hypothesize that the autologous fascia lata biological plug (FBP) represents a straightforward, cost-effective technique with a low risk of rejection, potentially leading to improved healing outcomes.

This study aims to assess the outcomes and complications associated with the management of high perianal fistulae using the FBP technique. The primary anticipated outcome is the healing of the fistulous tract, characterised by the absence of fistulous discharge, closure of the external opening, and the absence of perianal abscesses. Secondary outcomes include the patient's continence status and the time required for healing and closure of the fistula.

## Materials and methods

Ethical considerations

This study received approval from the Research Ethics Committee of the Faculty of Medicine at Cairo University, Cairo, Egypt. All enrolled patients provided written informed consent after being thoroughly counseled about the study's purpose and procedures. Participants' data confidentiality was assured.

Study design, setting, and period

This single-centre prospective cohort study was conducted at the General Surgery Department of Cairo University Hospitals in Egypt between March 2020 and December 2021.

Inclusion Criteria

Adult patients presenting to the Kasr Al-Ainy Outpatient Surgery Clinic with a single-tract high perianal fistula were included.

Exclusion Criteria

Patients with high perianal fistulas that exhibit side branches, active infections, a history of inflammatory bowel disease, and low anal fistulas, and those in vulnerable groups, such as pregnant patients, children, and cognitively impaired individuals, were excluded.

Procedure

Preoperative Assessment

All participants underwent a comprehensive medical history assessment, a thorough physical examination, imaging studies including an MRI of the anal canal to diagnose the type of fistula and identify any clinically unrecognised sepsis, and laboratory tests.

Preparation for the Operation

All patients underwent proper mechanical bowel preparation, which included clear fluids and a polyethylene glycol solution administered two days prior to surgery. Additionally, an enema was given the day before surgery and another on the morning of the procedure to ensure the absence of faecal material at the surgical site, thereby reducing the risk of wound infection. Prophylactic antibiotics (1 g of cefotaxime and 500 mg of metronidazole) were administered at the induction of anaesthesia, with dosages adjusted according to the patient's weight.

Steps of the Operation

**Figure 1:**
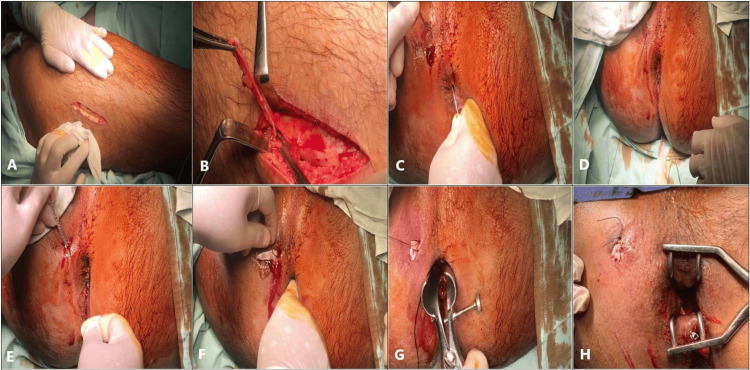
Fascia lata biological plug A - Lateral thigh incision, B - Closure of fascia lata, C - Passing the malleable probe, D - Passing silk suture in the tract, E - Curettage of the tract, F - Passing the plug in the tract, G - External fixation of the plug, H - fixation of the fascia lata plug at the external and internal openings

The patient was positioned in the lithotomy position under general or spinal anaesthesia. Following proper sterilisation, an anoscope was introduced to explore the anal canal and exclude any other pathologies. The internal and external openings were identified, and probing of the tract was performed using a malleable fistula probe to confirm the position of the fistula in accordance with our inclusion criteria. The patient was then repositioned supine, and a new Betadine draping was applied from the umbilicus to the below-the-knee joint on one side. An incision was made over the middle third of the lateral aspect of the thigh, one inch posterior to an imaginary line drawn from the anterior superior iliac spine to the lateral epicondyle of the femur, to expose the iliotibial band of the fascia lata. A segment of fascia lata measuring 4 inches in length and 1 inch in width was harvested, and the fascia defect was closed with interrupted Vicryl® 1 sutures. The subcutaneous and cutaneous layers were subsequently closed with interrupted sutures, and the wound was covered with a sterile dressing, with a crepe bandage applied over the lower limb. The harvested fascia was placed in a saline solution while preparing the fistulous tract for the insertion of the plug after modification of the fascia. The patient was repositioned in the lithotomy position with a new Betadine draping, and curettage of the tract was performed using a specialised fistula brush. Probing of the tract was conducted, connecting the plug with the probe through a silk suture, followed by the insertion of the plug into the tract. The plug was secured at both the internal and external openings using Vicryl® 2.0 sutures (Figure 1).

Post-operative Care

All patients were instructed to commence clear fluids two hours after recovery from anaesthesia for two days, followed by a low-residue diet for two weeks. They were prescribed oral antibiotics (ciprofloxacin 500 mg twice daily and metronidazole 500 mg every eight hours) for a duration of 10 days. Patients were advised to avoid using a water jet after defecation and to clean the area with a wet, clean towel instead. The donor site wound was covered during the operation and maintained with a clean dressing and crepe bandage for 10 days post-operatively. Subsequently, simple dressing changes were performed, and stitches were removed during follow-up at the outpatient clinic.

Follow-Up

All patients were followed up weekly at the Kasr Al-Ainy Outpatient Surgery Clinic for a duration of 18 months. During these follow-up visits, patients were questioned about their discharge, pain levels, and continence status. Continence was assessed using the Wexner score, which ranges from 0 to 20, where a score of 0 indicates perfect continence and a score of 20 signifies complete incontinence. Additionally, patients were examined for the presence of a fistulous opening and the condition of the thigh wound. Healing and cure of the fistula were defined as the complete closure of all openings and the absence of any discharge. Any patient with suspected recurrence identified during the clinical examination underwent an MRI for confirmation.

Statistical analysis

Data were coded and entered using Statistical Product and Service Solutions (SPSS, version 27; IBM SPSS Statistics for Windows, Armonk, NY). Quantitative variables were assessed for distribution using the Shapiro-Wilk test and were summarised using the mean and standard deviation for normally distributed data or the median and interquartile range for non-normally distributed data. The Mann-Whitney U test was applied to compare non-normally distributed data between two independent groups. Categorical data were presented as frequencies and percentages, with the chi-square test used for comparison. The exact test was applied when the expected frequency was less than five. P-values less than 0.05 were considered statistically significant.

## Results

In this study, we enrolled 46 patients, the majority of whom were male (37, 80.4%), resulting in a male-to-female ratio of 4:1. The mean age of the participants was 40.8±9.5 years, with an age range of 26-60 years (Table 1).

**Table 1:** Demographic characteristics of the studied patients (N=46) N: Number, SD: Standard deviation

Sex, N, %	Male	37 (80.4%)
	Female	9 (19.6%)
	Male:Female ratio	4:1
Age, year	Minimum	26.0
	Maximum	60.0
	Mean±SD	40.8±9.5

Among the 46 patients, seven (15.2%) had a history of diabetes mellitus, and a similar number reported a history of hypertension. Twenty patients (43.5%) had undergone abscess drainage, and 15 patients (32.6%) had an associated abscess cavity. Previous anal surgery was documented in 18 patients (39.1%). Regarding the types of fistulae, trans-sphincteric was the most common (58.7%), followed by inter-sphincteric (30.4%). Supra-sphincteric and extra-sphincteric types were less common (6.5% and 4.3%, respectively). The fistula was located posteriorly in 29 patients (63.0%), while it was positioned anteriorly in 17 patients (37.0%). The duration of the operation ranged from 15 to 50 minutes, with a mean duration of 30.7±8.9 minutes (Table 2).

**Table 2:** Preoperative and operative characteristics of the studied patients (N=46) N: Number, SD: Standard deviation

Preoperative and operative Characteristics	Finding	N=46	%
Comorbidities	No	32	69.6%
	Hypertension	7	15.2%
	Diabetes mellitus	7	15.2%
History of abscess drainage	No	26	56.5%
	Yes	20	43.5%
Associated abscess cavity	No	31	67.4%
	Yes	15	32.6%
Previous anal operation	No	28	60.9%
	Yes	18	39.1%
Type of anal fistula	Trans-sphincteric	27	58.7%
	Inter-sphincteric	14	30.4%
	Supra-sphincteric	3	6.5%
	Extra-sphincteric	2	4.3%
Site of anal fistula	Posterior	29	63.0%
	Anterior	17	37.0%
Duration of the operation, minutes	Minimum-maximum	15.0–50.0	
	Mean±SD	30.7±8.9	

The insertion of the FBP was associated with a success rate of 80.4% at six months post-operation, with nine patients (19.6%) experiencing recurrence of the fistula. The recurrence rate increased to 30.4% at 18 months post-operation, resulting in an overall success rate of 69.6%. All patients (100%) maintained complete continence. Healing of the fistula occurred within a duration ranging from four to nine weeks, with a mean healing time of 6.9±1.3 weeks. Follow-up of the thigh wound revealed infection in 10 patients (21.7%) (Table 3).

**Table 3:** Outcomes of the management of a high perianal fistula with a fascia lata biological plug N: Number, SD: Standard deviation

Outcome		N=46	%
Recurrence of the fistulous tract at six months (perianal wound infection)	No	37	80.4%
	Yes	9	19.6%
Recurrence of the fistulous tract at 18 months (perianal wound infection)	No	32	69.6%
	Yes	14	30.4%
Continence state	Complete continence	46	100.0%
Thigh wound infection	No	36	78.3%
	Yes	10	21.7%
Duration of healing, weeks	Minimum-Maximum	4.0–9.0	
	Mean±SD	6.9±1.3	

Table 4 demonstrates a significant association between the recurrence of fistulae at six months and a history of previous anal surgery (p=0.001). A substantial percentage (88.9%) of patients who experienced recurrence had undergone prior anal surgery. Additionally, the type of anal fistula showed a significant association with recurrence at six months (p=0.035), with higher recurrence rates observed in the extra-sphincteric and supra-sphincteric types.

**Table 4:** Factors associated with the recurrence of the fistulous tract at six months * Significant at p<0.05, IQR: Interquartile range

Factors		Recurrence of fistulous tract at six months				P-value
		No, N=37		Yes, N=9		
Age, years	Median (IQR)	40.0 (34.0–45.0)		55.0 (40.0–55.0)		0.087
Sex	Female	9	24.3%	0	0.0%	0.171
	Male	28	75.7%	9	100.0%	
Comorbidities	Diabetes mellitus	6	16.2%	1	11.1%	0.850
	Hypertension	5	13.5%	2	22.2%	
	No	26	70.3%	6	66.7%	
History of abscess drainage	No	23	62.2%	3	33.3%	0.149
	Yes	14	37.8%	6	66.7%	
Associated abscess cavity	No	24	64.9%	7	77.8%	0.696
	Yes	13	35.1%	2	22.2%	
Previous anal operation	No	27	73.0%	1	11.1%	0.001*
	Yes	10	27.0%	8	88.9%	
Type of anal fistula	Extra-sphincteric	0	0.0%	2	22.2%	0.035*
	Inter-sphincteric	13	35.1%	1	11.1%	
	Supra-sphincteric	2	5.4%	1	11.1%	
	Trans-sphincteric	22	59.5%	5	55.6%	
Site of anal fistula	Anterior	11	29.7%	6	66.7%	0.058
	Posterior	26	70.3%	3	33.3%	

The analysis of factors associated with treatment failure and recurrence at 18 months revealed a significantly higher recurrence rate for extra-sphincteric (14.3% vs 0.0%) and supra-sphincteric (21.4% vs 0.0%) types (p=0.006). Anterior fistulae also exhibited a significantly higher recurrence rate compared to posterior fistulae (64.3% vs 25.0%, p=0.011) (Table 5).

**Table 5:** Factors associated with the recurrence of a fistulous tract at 18 months * Significant at p<0.05, IQR: Interquartile range

Factors		Recurrence of fistulous tract at 18 months				P-Value
		No N=32		Yes N=14		
Age, years	Median (IQR)	40.0 (35.0–45.0)		36.5 (30.0–55.0)		0.755
Sex	Female	6	18.8%	3	21.4%	1.00
	Male	26	81.3%	11	78.6%	
Comorbidities	Diabetes mellitus	4	12.5%	3	21.4%	0.158
	Hypertension	7	21.9%	0	0.0%	
	No	21	65.6%	11	78.6%	
History of abscess drainage	No	21	65.6%	5	35.7%	0.060
	Yes	11	34.4%	9	64.3%	
Associated abscess cavity	No	21	65.6%	10	71.4%	1.00
	Yes	11	34.4%	4	28.6%	
Previous anal operation	No	21	65.6%	7	50.0%	0.318
	Yes	11	34.4%	7	50.0%	
Type of anal fistula	Extra-sphincteric	0	0.0%	2	14.3%	0.006*
	Inter-sphincteric	11	34.4%	3	21.4%	
	Supra-sphincteric	0	0.0%	3	21.4%	
	Trans-sphincteric	21	65.6%	6	42.9%	
Site of anal fistula	Anterior	8	25.0%	9	64.3%	0.011*
	Posterior	24	75.0%	5	35.7%	

## Discussion

Anal fistulae present a significant challenge for both surgeons and patients. The risk of frequent recurrence and incontinence poses a considerable threat to the long-term management of anal fistulae. Consequently, surgeons are continually developing new techniques to manage anal fistulae without compromising the patient's continence [14].

In the present study, the insertion of the FBP resulted in complete healing without recurrence in 37 patients (80.4%) at six months post-operation. Among the 46 patients, only nine (19.6%) experienced a recurrence of the fistula. The recurrence rate increased to 30.4% at 18 months post-operation, yielding an overall success rate of 69.6%. These findings are comparable to the reported success rates for the Surgisis® AFP and the Gore BIO-A fistula plug for high anal fistulae. The initial evaluation of the Surgisis® plug by Champagne et al. [15] revealed a success rate of 87% at a mean follow-up of two years. Subsequent studies on the Surgisis® plug reported variable healing rates, ranging from 43% [16] to 86% [17]. A larger study involving 51 patients demonstrated complete healing in 23 patients (56.1%) during a 12-month follow-up period [18]. Limura et al. [10], in their review, reported variable success rates for the Cook Surgisis® AFP, ranging from 24% to 88%, with a mean follow-up of eight months.

Earlier studies evaluating the Gore BIO-A fistula plug for complex high trans-sphincteric anal fistulae revealed a success rate of 72.7% at five months [19]. Conversely, the closure of high trans-sphincteric or supra-sphincteric fistulae showed a lower success rate of 57.5% (23/40) [20]. A case series involving 12 patients treated with the Gore BIO-A fistula plug for high trans-sphincteric fistulae demonstrated a high success rate (83.4%), with only two patients (16.6%) experiencing recurrence at two and three months, respectively [21]. Additionally, a systematic review encompassing six studies using the Gore BIO-A® plug indicated that the fistula healing rate varied from 15.8% to 72.7% over follow-up periods ranging from two to 19 months [22].

Various risk factors can impair the healing of anal fistulae. A long-term follow-up study on the AFP technique conducted over eight years identified the primary reasons for treatment failure as plug extrusion (20%) and surgical site infection within 30 days post-surgery (15%) [9]. In the present study, a history of previous anal surgery significantly impacted the success rate of fistula healing. This finding suggests that patients with fewer prior procedures are more likely to benefit from FBP insertion. Scarring and fibrosis resulting from previous anal surgeries may contribute to the early dislodgement of the fistula plug, as reported by Thekkinkattil et al. [23], or hinder the healing of fistulae, as suggested by Sentovich [24].

The present study also indicated a significantly increased risk of recurrence associated with extra-sphincteric and supra-sphincteric fistulae compared to trans-sphincteric and inter-sphincteric types. The anatomy of the fistula is a known risk factor for recurrence, with supra-sphincteric and extra-sphincteric types associated with a higher likelihood of complications and recurrence [25]. Khadia et al. [26] reported that all patients with a supra-sphincteric fistula experienced recurrence, with this type contributing to 39% of all fistula types studied.

Furthermore, the study revealed that anterior fistulae were significantly associated with a higher recurrence rate at 18 months post-surgery. This finding aligns with the research conducted by Taema et al. [27], who investigated seton placement in 50 patients with high trans-sphincteric perianal fistula and identified anterior anal fistulae as significant predictors of recurrence. Additionally, Emile et al. [28] showed a 3.36-fold increase in the likelihood of recurrence associated with the anterior fistula type following seton placement for high trans-sphincteric fistulae.

One of the primary objectives of anal fistula surgery is to preserve the patient's anal sphincter function, thereby ensuring a good quality of life. Follow-up assessments of patients after fascia lata plug placement in this study revealed complete continence in all cases, with no adverse effects on faecal continence. Similarly, Herold et al. [29] demonstrated that the use of the Gore BIO-A synthetic plug in the treatment of anal fistula did not compromise continence. Heydari et al. [8] also reported that none of the patients treated with bioabsorbable synthetic plugs experienced post-operative faecal incontinence at three, six, or 12 months post-evaluation. Furthermore, a meta-analysis involving 408 patients indicated a lower rate of post-operative incontinence with the AFP compared to the mucosal advancement flap procedure [30]. Alternatively, long-term follow-up over eight years after using the AFP for trans-sphincteric fistula revealed decreased anal function in 33 (26.8%) patients after surgery [31].

The mean duration of the operation using our novel technique was 30.7 minutes, with a range of 15-50 minutes, reflecting improvements in technique over time. This duration is comparable to the inter-sphincteric fistula tract ligation technique, which had a median duration of 35 minutes, as reported by Lehmann et al. [32]. However, it is considered more time-consuming than the LASER technique, which averages only 18.3±7.9 minutes [33].

This study has several limitations. Firstly, it is a single-institution prospective case series that lacks a control group. Secondly, the diagnosis of fistula healing and cure was based on subjective assessments, and we did not perform magnetic resonance imaging for confirmation. However, due to financial constraints, achieving discharge and clinical cure were prioritised over radiological healing. Further multicentre randomised clinical trials with longer follow-up periods are needed to compare different AFPs for high anal fistulae.

## Conclusions

The FBP represents a promising sphincter-sparing technique. The insertion of the FBP for single-tract high perianal fistulae has demonstrated favourable outcomes without compromising patients' continence. The recurrence rate was 19.6% at six months, increasing to 30.4% at 18 months. A history of previous anal surgery, along with extra-sphincteric and supra-sphincteric types, as well as anterior fistulae, was associated with a higher recurrence rate. It is essential to select the procedure based on the type and nature of the anal fistula to ensure success.
